# Acute Compartment Syndrome of the Arm after Minor Trauma in a Patient with Optimal Range of Oral Anticoagulant Therapy: A Case Report

**DOI:** 10.1155/2014/980940

**Published:** 2014-01-06

**Authors:** Paolo Titolo, Patrizia Milani, Bernardino Panero, Davide Ciclamini, Giulia Colzani, Stefano Artiaco

**Affiliations:** Department of Orthopaedics, Traumatology and Rehabilitation, AO Città della Salute e della Scienza, Orthopaedic and Trauma Center, Via Zuretti 29, 10126 Turin, Italy

## Abstract

Compartment syndrome of the arm is a rare event that can be subsequent to trauma or other pathological and physical conditions. At the arm the thin and elastic fascia may allow accumulation of blood more than in other districts, especially in patients undergoing anticoagulant therapy. We describe a rare case of an acute compartment syndrome of the arm after minor trauma with partial biceps brachii rupture in a patient with warfarin therapy and optimal value of INR. Prompt diagnosis and surgical decompression helped to avoid the occurrence of complications with a satisfying recovery of arm function.

## 1. Introduction

Compartment syndrome of the arm is rare. In most cases, it occurs after trauma [1, 2], biceps or triceps rupture [3], subatmospheric pressure exposition [4], tourniquets application [5], shoulder dislocation [6], surgical complication [7], blood pressure cuff malpositions [8], injections [9], and venipunctures [10, 11].

Arm fascia is relatively thinner and more elastic compared to other anatomical districts. This creates more space for accumulation of blood. Variability of tolerance to increased intracompartmental pressure may influence clinical onset and the course of the syndrome [12, 13].

Patients undergoing anticoagulant therapy raise their susceptibility to haemorrhage, and sometimes these drugs can facilitate the onset of an acute compartment syndrome. The relationship between acute compartment syndrome and warfarin treatment was first described by Hay et al. in 1992 in seven patients who sustained minor trauma in lower limbs (six cases) and forearm (one case) [14].

In this report, we describe a case of acute compartment syndrome of the arm after minor trauma with partial rupture of short head of biceps brachii in a patient undergoing warfarin therapy. To our knowledge this is the first case reported in the literature in which such kind of syndrome occurred in a patient with optimal value of INR.

## 2. Case Report

A 66-year-old right hand dominant woman reported minor shoulder trauma with arm elongation. She was assuming warfarin for 15 years after aortic valve replacement. At the time of hospital admission, she presented with pain and swelling at the right arm. A wide hematoma was present from the axillary region to the anteromedial side of the arm and the patient complained of limitation and pain of right upper arm during movements without vascular and nervous impairments. Laboratory values revealed increased levels of WBC (12.5 × 1000/*μ*L), Hb 13.2 g/dL, APTT 75.8 sec, LDH 735 UI/L, CK 103 UI/L, and INR of 3.0. Radiographs did not show fractures or dislocations. The ultrasound detected an intramuscular haematoma (3.5 cm in diameter, 15 cm in length) in the short head of biceps brachii without joint effusion.

Patient was admitted for monitoring and observation. Warfarin was stopped and low molecular weight heparin (LMWH) was given [15]. During the first day of observation the patient complained of increasing pain and laboratory exams showed further increase of WBC (19.5 × 1000/*μ*L) with INR 2. Two days after admission the ecchymosis was extended to the proximal third of the arm and radial and ulnar nerve palsy was observed. The median nerve was uninvolved and peripheral arterial pulses were present. The intracompartmental pressure measured with STRYKER 295-2 QUICK PRESSURE MONITOR SET was 40 mmHg. Laboratory test values revealed WBC 20.6 × 1000/*μ*L, Hb 11.8 g/dL, Hct 34.6%, INR 1.41, APTT 41.8 sec LDH 715 UI/L, CK 736 UI/L, myoglobin 540 ng/mL.

Surgical treatment was planned with fasciotomy and drainage of haematoma through a deltoid-pectoral approach extended to the medial part of the upper arm. At the opening of brachial fascia, muscles appeared strained and compressed. The septum among brachialis and biceps muscle was opened and the haematoma was drained. Wound was closed with interrupted suture and a noncompressive bandage was applied. Postoperative therapy was prescribed giving disodium phosphate betamethasone, L-acetyl carnitine, paracetamol, and tramadol.

One day after surgery laboratory values revealed decreased WBC (12.6 × 1000/*μ*L), INR 1.13, APTT 29.6, myoglobin 334.70 ng/mL. During postoperative period the patient started rehabilitation program. When the patient was discharged, he presented with limitation of hand and fingers movements and numbness for residual involvement of the ulnar nerve.

Four months after surgery, the patient showed almost complete triceps function (4/5 according to Medical Research Council scale) and full recovery of elbow flexion and wrist and finger motion. Sensibility was normal with two-point discrimination test value less than 5 mm. Residual stiffness of the shoulder in abduction and external rotation was observed.

One year after surgery electroneurography showed normal radial, median, and ulnar nerves function and the patient reported a slight residual fatigue in the activity of daily living.

## 3. Discussion

Patients undergoing anticoagulant therapy raise their susceptibility to haemorrhage and are more exposed to development of an acute compartment syndrome.

Acute compartment syndrome after biceps brachii ruptures has been described in the literature in two patients with uncontrolled high INR value secondary to anticoagulant therapy. Richards reported a case of biceps long head rupture resulting in an upper arm compartment syndrome associated with nerve palsies in a patient who presented with INR value of 7.1 at hospital admission [16]. Another case of acute arm compartment syndrome was reported by Fung in a patient with biceps tendon rupture and an INR of 6.5 at time of clinical observation [17]. Only one compartment syndrome of the arm following minor trauma was previously reported in the literature in a patient assuming warfarin [18]. In our patient, the syndrome was diagnosed two days after trauma although the INR value was in controlled therapeutic range.

In patients with warfarin therapy, the risk of development of compartment syndrome should be always considered and prolonged clinical observation should be indicated in order to detect initial clinical symptoms. Early treatment with fasciotomy may prevent major complications and residual deficits.
